# Correlation between stress hyperglycemia ratio and prognosis in acute myocardial infarction patients following percutaneous coronary intervention

**DOI:** 10.3389/fcvm.2025.1493635

**Published:** 2025-05-09

**Authors:** Wei Meng, Hui Qiu, Weiping Li, Hongwei Li

**Affiliations:** ^1^ Department of Cardiology, Cardiovascular Center, Beijing Friendship Hospital, Capital Medical University, Beijing, China; ^2^ Beijing Key Laboratory of Metabolic Disorders Related Cardiovascular Disease, Beijing, China; ^3^ Laboratory for Clinical Medicine, Capital Medical University, Beijing, China

**Keywords:** acute myocardial infarction, major adverse cardiovascular and cerebrovascular events, percutaneous coronary intervention, stress hyperglycemia, stress hyperglycemia ratio

## Abstract

**Background:**

Stress hyperglycemia ratio (SHR) is a commonly used predictor of acute hyperglycemia. The present study aimed to evaluate the prognostic significance of SHR in acute myocardial infarction (AMI) patients who underwent percutaneous coronary intervention (PCI).

**Methods:**

A total of 3,212 consecutive AMI patients who underwent PCI were recruited and assigned to three groups, according to SHR tertiles. Then, the total number of major adverse cardiovascular and cerebrovascular events (MACCEs) and various cardiovascular events were recorded. The SHR was determined, as follows: admission blood glucose (mmol/L)/[1.59 × hemoglobin A1c (%) −2.59].

**Results:**

The incidence of MACCEs was positively correlated to SHR during the median follow-up of 36 months. The multivariate COX regression analysis identified SHR as an independent predictor of composite MACCEs [hazard ratio: 2.279, 95% confidence interval (CI): 1.569–3.311, *p* < 0.001] and target vessel revascularization (hazard ratio: 1.998, 95% CI: 1.299–3.074, *p* = 0.002). In terms of gender, age, type of AMI, body mass index, left ventricular ejection fraction, and diabetes mellitus, SHR >1.45 was significantly associated to MACCEs across all subgroups (all, *p* < 0.001), except for patients with ejection fraction <50%. Furthermore, the area under the receiver operating characteristic curve for SHR in predicting MACCEs was 0.636 (95% CI: 0.613–0.659, *p* < 0.05), with a cut-off value of 1.317.

**Conclusions:**

Stress hyperglycemia, as indicated by SHR, is significantly correlated to MACCEs, and independently predicts the prognosis of AMI patients undergoing PCI. These findings highlight the potential of SHR as an effective prognostic marker for AMI patients undergoing PCI.

## 1 Introduction

Acute myocardial infarction (AMI) remains as the leading cause of death globally, despite the significant advancements in primary percutaneous coronary intervention (PCI) and pharmacotherapy over the past decade (1). Thus, identifying risk factors for the poor prognosis of AMI patients following PCI is of great importance. Stress hyperglycemia refers to the metabolic disturbance that occurs during stress conditions, such as severe infection, trauma, excessive bleeding, or acute poisoning. Furthermore, this is characterized by disruptions in glucose metabolism. As a severe stressor on the body, AMI can trigger stress hyperglycemia (2). Epidemiological evidence has indicated that approximately 25%–50% of patients with acute coronary syndrome (ACS) may experience stress hyperglycemia (3). This acute physiological response to stress may destabilize and rupture atherosclerotic plaques, accelerate myocardial ischemia (4, 5), and become a strong predictor of mortality in critically ill patients (6, 7).

For patients with ACS, particularly for patients with AMI, stress hyperglycemia is independently associated to poor long-term and short-term outcomes (8, 9). However, there is no universally accepted definition of stress hyperglycemia for patients with AMI, and its predictive value remains controversial. Discrepancies in the study results may be attributed to the use of admission glucose levels, as a sole measure of stress hyperglycemia. Since absolute admission glucose concentrations can be affected by both chronic elevated baseline glucose and acute physiological stress, these may not always accurately reflect the true level of stress hyperglycemia (10). Thus, the stress hyperglycemia ratio (SHR), which is defined as the ratio of admission blood glucose (ABG) to the estimated average glucose, was introduced to address this issue (11). SHR has been shown to provide superior predictive value for cases with AMI, when compared to ABG alone (12, 13).

Most investigations on the relationship between stress hyperglycemia and AMI have primarily focused on ST-segment elevation myocardial infarction (STEMI). For instance, SHR was found to be independently associated to cardiac function and microvascular obstruction in patients who underwent primary PCI for acute STEMI (14). In addition, a previous study identified SHR as an independent predictor for in-hospital major adverse cardiovascular and cerebrovascular events (MACCEs) in acute STEMI patients, especially for patients without diabetes (15). Furthermore, SHR can independently predict in-hospital heart failure in patients with anterior STEMI (16). However, the predictive value of stress hyperglycemia across different subgroups of patients remain controversial. A multi-center nationwide registry study revealed that SHR was significantly positively correlated to increased risk of both all-cause and cardiovascular mortality in patients with coronary artery disease (CAD) and chronic kidney disease, while no such association was observed in CAD patients without chronic kidney disease (17). Furthermore, SHR was an independent predictor for hospitalization risk in patients with ischemia and non-obstructive coronary arteries, regardless of the diabetes status (18).

Therefore, the present study aimed to determine the correlation between SHR and adverse outcomes in AMI patients who underwent PCI, and determine whether these correlations differ among various subgroups.

## 2 Methods

### 2.1 Study participants

**Figure 1:**
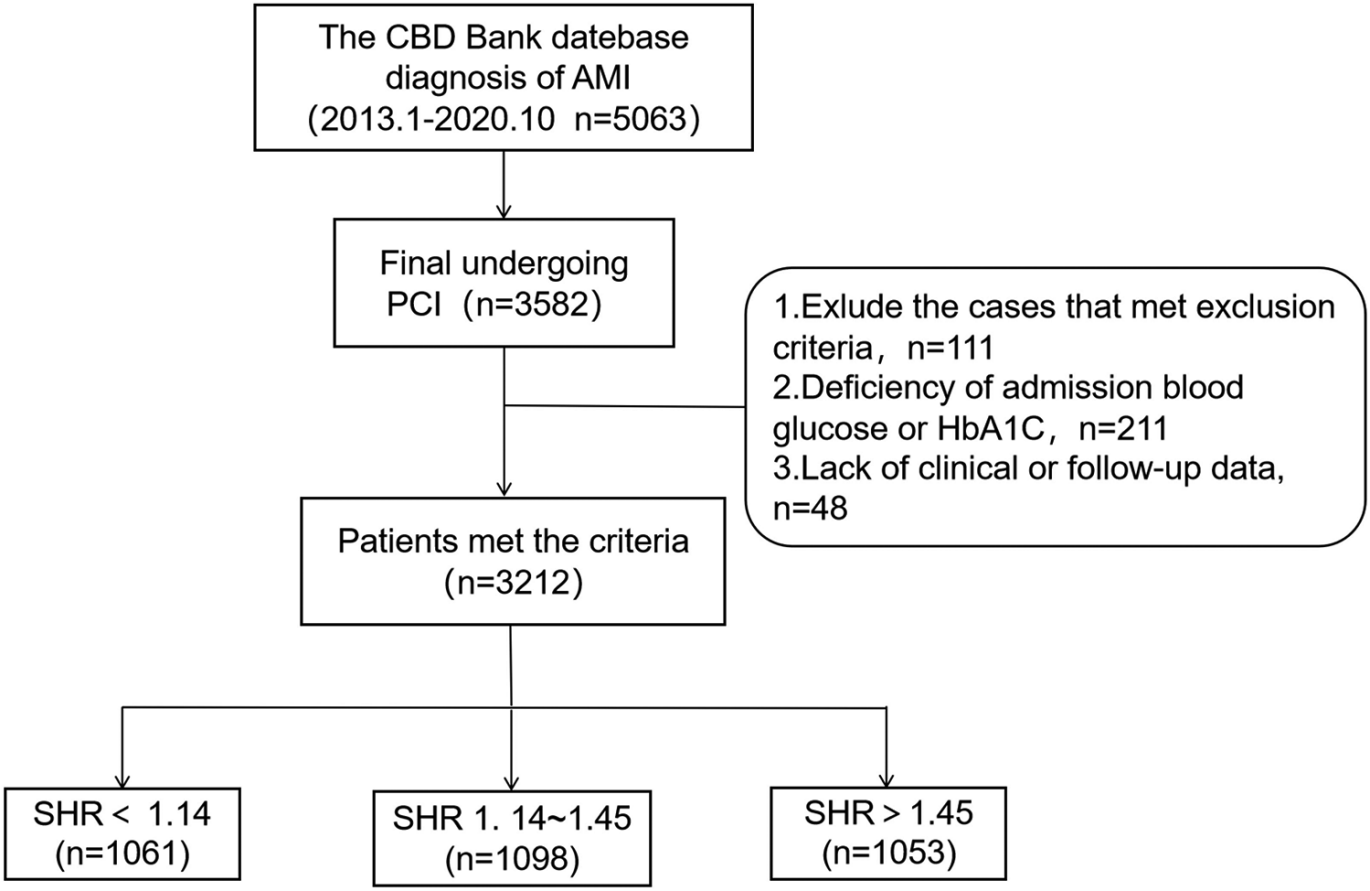
Flowchart for the enrollment of subjects. CBD, Cardiovascular Center of Beijing Friendship Hospital Database; PCI, percutaneous coronary intervention; SHR, stress hyperglycemia ratio.

For the present retrospective, observational study, participants were recruited from the Cardiovascular Center of Beijing Friendship Hospital Database Bank. The enrollment process is presented in Figure 1. A total of 5,063 consecutive patients, who were diagnosed with AMI from January 2013 to October 2020, were screened. Inclusion criteria: (1) age ≥18 years old; (2) patients diagnosed with AMI; (3) patients who met the criteria for PCI, and underwent emergency or elective PCI during hospitalization based on the clinical condition. Exclusion criteria: (1) severe structural heart disease, severe valvulopathy, or cardiomyopathy; (2) severe hepatic dysfunction, kidney transplantation, eGFR <30 ml/min/1.73 m^2^, or chronic dialysis; (3) hemoglobin <60 g/L, malignant tumor, hematological disease, or serious infections with a life expectancy of <12 months; (4) missing important laboratory data, such as admission blood glucose and glycated hemoglobin (HbA1c) levels, or lacking follow-up data. Next, these patients were grouped according to tertiles of SHR: SHR < 1.14 group, 1.14 ≤ SHR ≤ 1.45 group, and SHR > 1.45 group. Coronary angiography and related procedures were performed in strict accordance to present guideline recommendations. Antiplatelet and perioperative anticoagulation therapies were administered following standardized protocols. After discharge, all patients received guideline-directed secondary prevention medications for CAD.

### 2.2 Data collection and definitions

Demographic information, medical history, laboratory test results, medical treatment, and angiographic and echocardiographic evaluation results were obtained from all patients. Then, the MACCEs were documented and recorded during the clinical follow-up visits. Each patient was followed up at 1, 3, 6, and 12 months through outpatient clinic visits or telephone questionnaires. Subsequently, annual follow-ups were conducted.

The SHR was calculated, as follows: admission blood glucose (mmol/L)/[1.59 × HbA1c (%) - 2.59]. AMI included both non-STEMI and STEMI, which was defined as chest pain with newly detected ST-segment alterations, and elevation in myocardial necrosis markers to more than twice the upper limit of the normal range. The primary endpoint was the MACCE at follow-up, which included all-cause death, recurrent myocardial infarction (MI), cardiovascular death (CV death), target vessel revascularization, readmission for heart failure, and stroke. All-cause death was defined as mortality due to any cause, which comprised of both cardiac and non-cardiac origins. CV death refers to death caused by any heart disease. Target vessel revascularization refers to the revascularization of the target coronary artery through PCI (including both emergency and elective procedures) or coronary artery bypass grafting (CABG). Stroke was characterized as having a history of transient ischemic attacks, ischemic stroke, or cerebral hemorrhage.

### 2.3 Statistical analysis

The statistical analysis was conducted using IBM SPSS 23.0. Continuous data are expressed in mean ± standard deviation (SD) or median [interquartile range (IQR)]. Comparisons between the three groups were made using the Kruskal–Wallis test or one-way ANOVA. Categorical data were presented in numbers and percentages, and compared using Fisher's exact test or Pearson's Chi-square test. Kaplan–Meier survival curves were used to analyze the cumulative incidence of MACCEs, with group comparisons performed by log-rank test. The baseline and clinical characteristics that were significantly correlated to MACCEs in the univariate analysis were included in the multivariate model. Multivariate Cox regression analysis was performed to determine whether SHR can independently predict MACCEs, and identify other potential predictors. Receiver operating characteristic (ROC) curves were used to determine the optimal cut-off value for SHR in predicting MACCEs. A *p*-value of <0.05 indicated statistical significance, with 95% confidence interval (CI).

## 3 Results

### 3.1 Baseline characteristics

A total of 3,582 patients underwent PCI. Among these patients, 370 patients were excluded based on the exclusion criteria, resulting in 3,212 patients who met the selection criteria for analysis. All patients were followed up until September 31, 2021, with a median follow-up duration of 36 months (IQR: 13–60 months). Then, these patients were classified into three groups based on the SHR: SHR < 1.14 group (*n* = 1,061), 1.14 ≤ SHR ≤ 1.45 group (*n* = 1,098), and SHR > 1.45 group (*n* = 1,053). Patients with SHR >1.45 exhibited higher rates of STEMI, diabetes mellitus (DM), and the prehospital use of antidiabetics, and higher TyG index levels, when compared to the other two groups (*p* < 0.001). Furthermore, these patients had higher rates of hypertension (*p* = 0.015). Moreover, the white cell count, admission plasma glucose (ABG), fasting plasma glucose (FPG), HbA1c, *Δ*A-C {which was calculated, as follows: ABG - [1.59 × HbA1c (%) - 2.59]}, high-sensitivity C-reactive protein, peak of N-terminal pro-brain natriuretic peptide (pNT-proBNP), peak of cardiac troponin I, and peak of creatine kinase isoenzyme MB were the highest in the SHR > 1.45 group, and the lowest in the SHR < 1.14 group (*p* < 0.01, Table 1).

**Table 1:** Baseline characteristics of patients stratified by tertiles of SHR.

Variables	SHR < 1.14 (*n* = 1,061)	1.14 ≤ SHR ≤ 1.45 (*n* = 1,098)	SHR > 1.45 (*n* = 1,053)	*p*-value
Male gender	816 (78.00)	866 (77.50)	782 (74.50)	0.124
Age, years	62.93 ± 12.17	62.85 ± 11.81	63.45 ± 11.47	0.444
BMI, kg/m^2^	25.51 ± 3.48	25.48 ± 3.72	25.66 ± 3.65	0.454
SBP, mmHg	130.46 ± 21.65	127.97 ± 20.84	128.69 ± 22.83	0.025*
DBP, mmHg	74.11 ± 12.52	73.44 ± 12.54	73.70 ± 12.90	0.473
Heart rate, bpm	74.13 ± 14.37	75.01 ± 15.22	75.66 ± 15.67	0.065
Current/ex-Smoker	680 (64.09)	684 (62.30)	655 (62.20)	0.597
STEMI	528 (49.76)	615 (56.01)	639 (60.68)	<0.001**
TyG index	8.78 ± 0.63	8.91 ± 0.66	9.18 ± 0.75	<0.001**
Medical history				
Hypertension	677 (63.81)	698 (63.57)	725 (68.85)	0.015*
Dyslipidemia	511 (48.16)	528 (48.09)	535 (50.81)	0.361
Diabetes mellitus	353 (33.27)	345 (31.42)	554 (52.61)	<0.001**
IFG	106 (10.00)	166 (15.10)	127 (12.10)	0.001**
Stoke	149 (14.04)	178 (16.21)	178 (16.90)	0.168
OMI	142 (13.38)	114 (10.38)	96 (9.12)	0.005**
PCI	147 (13.85)	153 (13.93)	125 (11.87)	0.282
CABG	23 (2.17)	18 (1.64)	18 (1.71)	0.614
Heart failure	12 (1.13)	11 (1.00)	10 (0.95)	0.912
Chronic kidney diseases	44 (4.15)	55 (5.01)	50 (4.75)	0.622
Medication used before admission				
Antiplatelet agent	314 (29.59)	298 (27.14)	300 (28.49)	0.448
ACEI/ARB	290 (27.33)	290 (26.41)	265 (25.17)	0.525
Beta-blocker	144 (13.57)	143 (13.02)	153 (14.53)	0.591
Stain	148 (13.95)	144 (13.11)	158 (15.00)	0.450
Antidiabetics	236 (22.24)	277 (25.23)	385 (36.56)	<0.001**
Angiography findings				
Multivessel/LMCA	322 (37.30)	324 (37.00)	360 (41.50)	0.100
Proximal LAD	371 (43.00)	387 (44.20)	391 (45.20)	0.676
TIMI score	4.20 (0.70)	4.49 (0.69)	4.60 (0.73)	<0.001**
Laboratory values				
WBC, 10^9^/L	8.18 ± 2.74	8.64 ± 2.94	9.24 ± 3.26	<0.001**
ABG, mmol/L	6.15 ± 1.21	8.10 ± 1.90	13.18 ± 4.55	<0.001**
FPG, mmol/L	5.27 (4.80, 6.00)	5.78 (5.00, 6.90)	7.62 (5.80, 10.30)	<0.001**
HbA1c, %	6.25 ± 1.23	6.31 ± 1.36	7.22 ± 1.81	<0.001**
ΔA-C, mmol/L	−0.87 (−1.60, −0.40)	0.69 (0.20, 1.10)	3.38 (2.40, 5.30)	<0.001**
Albumin, g/L	37.15 ± 3.97	37.42 ± 3.78	37.04 ± 3.99	0.075
Uric acid, umol/L	356.70 ± 92.41	342.59 ± 98.18	340.94 ± 103.25	<0.001**
Hemoglobin, g/L	135.55 ± 18.19	136.93 ± 18.46	136.63 ± 18.55	0.196
Creatinine, umol/L	81.70 (71.60, 94.10)	79.20 (69.10, 91.80)	79.30 (68.60, 94.20)	0.008**
eGFR, ml/min/1.73 m^2^	82.41 ± 23.61	84.68 ± 22.49	82.19 ± 24.30	0.026*
TC, mmol/L	4.47 ± 1.07	4.57 ± 1.07	4.44 ± 1.11	0.021*
TG, mmol/L	1.38 (1.00, 2.00)	1.45 (1.10, 2.10)	1.44 (1.10, 2.10)	0.078
LDL-C, mmol/L	2.62 ± 0.77	2.66 ± 0.77	2.58 ± 0.80	0.066
HDL-C, mmol/L	1.01 ± 0.24	1.05 ± 0.27	1.04 ± 0.24	0.001**
D-Dimer, ug/ml	0.60 (0.40, 0.80)	0.60 (0.40, 0.90)	0.60 (0.40, 0.90)	0.745
hs-CRP, mg/L	36.82 ± 53.24	32.49 ± 49.12	40.34 ± 54.41	0.003**
LVEF, %	0.59 ± 0.10	0.58 ± 0.10	0.58 ± 0.10	<0.001**
pNT-proBNP, pg/ml	3,536.26 ± 6,125.66	4,050.98 ± 6,625.45	4,672.23 ± 7,437.70	0.001**
pMyo, ng/ml	85.50 ± 122.46	116.70 ± 148.84	111.49 ± 141.07	<0.001**
pCK-MB, ng/ml	142.42 ± 209.13	176.33 ± 243.36	191.22 ± 255.65	<0.001**
pTNI, ng/ml	9.54 ± 13.94	12.61 ± 16.21	13.07 ± 16.37	<0.001**
In-hospital treatment				
Primary PCI	293 (27.62)	453 (41.26)	463 (43.97)	<0.001**
Antiplatelet agent	999 (94.16)	1,035 (94.26)	998 (94.78)	0.802
ACEI/ARB	705 (66.45)	699 (63.66)	701 (66.57)	0.273
β-blocker	762 (71.82)	798 (72.68)	786 (74.64)	0.324
Stain	932 (87.84)	951 (86.61)	929 (88.22)	0.495
Antidiabetics	249 (23.47)	278 (25.32)	429 (40.74)	<0.001**

The data are presented in mean ± standard deviation (SD), median [interquartile range (IQR)], or number (%). SHR, stress hyperglycemia ratio; BMI, body mass index; SBP, systolic blood pressure; DBP, diastolic blood pressure; STEMI, ST-segment elevation myocardial infarction; TyG, triglyceride-glucose; IFG, impaired fasting glucose; OMI, old myocardial infarction; PCI, percutaneous coronary intervention; CABG, coronary artery bypass graft; ACEI/ARB, angiotensin-converting enzyme inhibitor/angiotensin receptor blocker; LMCA, left main coronary artery; LAD, left anterior descending; TIMI, thrombolysis in myocardial infarction; WBC, white blood cells; ABG, admission plasma glucose; FPG, fasting plasma glucose; HbA1c, glycated hemoglobin; ΔA-C, ABG—[1.59 × Hb A1c (%) −2.59]; eGFR, estimated glomerular filtration rate; TC, total cholesterol; TG, triglyceride; LDL-C, low-density lipoprotein cholesterol; HDL-C, high-density lipoprotein cholesterol; hs-CRP, high-sensitivity C reactive protein; LVEF, left ventricular ejection fraction; pNT-proBNP, peak of N-terminal pro-brain natriuretic peptide; pCK-MB, peak of creatine kinase isoenzyme MB; pTNI, peak of cardiac troponin I.

* *p* < 0.05.

** *p* < 0.01.

The SHR > 1.45 group had the highest, while the SHR < 1.14 group had the lowest proportion of emergency PCI (*p* < 0.001). However, the SHR < 1.14 group had the highest rates of systolic blood pressure and old MI (*p* < 0.05). Furthermore, these patients presented with significantly higher creatinine levels and ejection fraction (EF) values measured by echocardiography, when compared to the other two groups (*p* < 0.01). In addition, the proportion of impaired fasting glucose (IFG) was significantly higher in the 1.14 ≤ SHR ≤ 1.45 group, when compared to the SHR < 1.14 group (*p* < 0.001). Moreover, the use of antidiabetics during hospitalization was higher in the SHR > 1.45 group, when compared to the other two groups (*p* < 0.001), while the use of other medications did not significantly differ among the three groups (Table 1).

### 3.2 Kaplan–Meier survival analysis for cardiovascular outcomes

**Figure 2:**
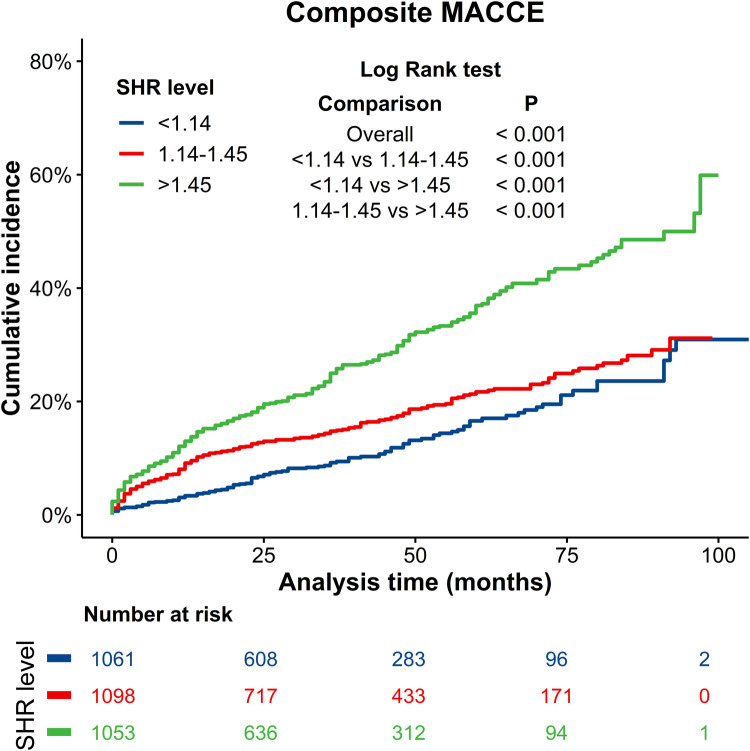
Kaplan–Meier curves for composite MACCEs. Blue line: SHR < 1.14 group; Red line: 1.14 ≤ SHR ≤ 1.45 group; Green line: SHR > 1.45 group. MACCEs, major adverse cardiac and cerebrovascular events.

The median follow-up duration was 36 months. During this period, a total of 655 (20.4%) patients experienced MACCEs, which included 290 (9.00%) cases of all-cause death, 146 (4.50%) cases of CV death, 216 (6.70%) cases of recurrent MI, 318 (9.90%) cases of target vessel revascularization, 109 (3.40%) cases of readmission for heart failure, and 56 (1.70%) cases of stroke. Composite MACCEs occurred in 121 (11.40%) patients in the SHR < 1.14 group, 208 (18.94%) patients in the 1.14 ≤ SHR ≤ 1.45 group, and 326 (30.96%) patients in the SHR > 1.45 group. The cumulative incidence curve for MACCEs in patients with different SHR levels is presented in Figure 2. The incidence of MACCEs was significantly higher in the SHR > 1.45 group, when compared to the SHR < 1.14 and 1.14 ≤ SHR ≤ 1.45 groups (*p* < 0.001).

**Figure 3:**
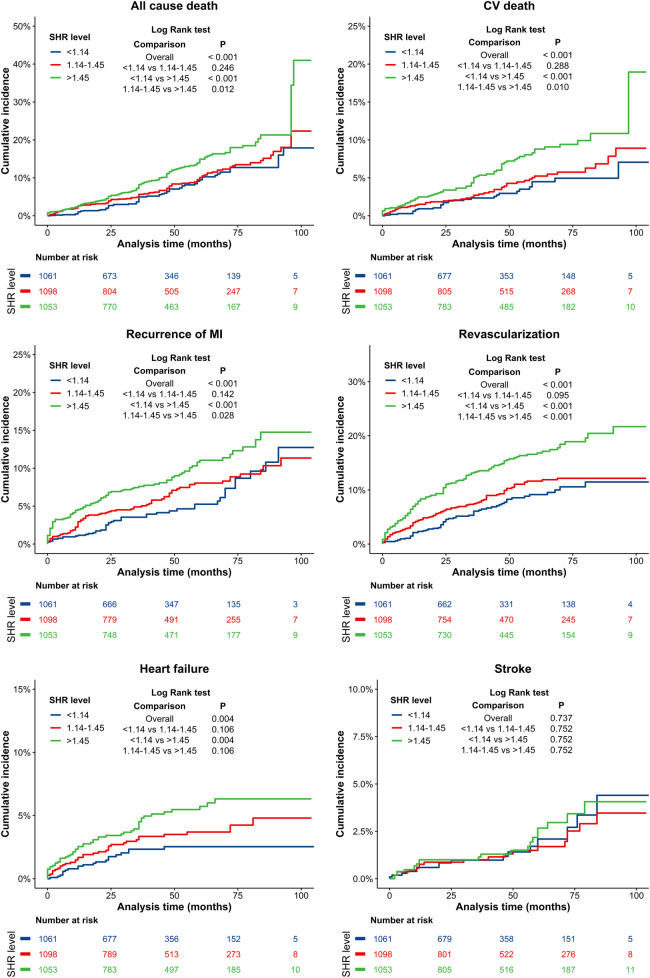
Kaplan–Meier curves for all-cause death, CV death, recurrence MI, revascularization, heart failure, and stroke. Blue line: SHR < 1.14 group; Red line: 1.14 ≤ SHR ≤ 1.45 group; Green line: SHR > 1.45 group. CV, cardiovascular; MI, myocardial infarction.

The cumulative incidence curves for all-cause mortality, CV death, recurrent MI, revascularization, heart failure, and stroke in patients with different SHR levels are presented in Figure 3. There were no significant differences in follow-up stroke incidence among the three groups of SHR patients (*p* > 0.05). However, the SHR > 1.45 group had significantly higher incidences of all-cause death, CV death, recurrent MI and revascularization events, when compared to the SHR < 1.14 and 1.14 ≤ SHR ≤ 1.45 groups (*p* < 0.05), while no significant differences were observed between the 1.14 ≤ SHR ≤ 1.45 and SHR < 1.14 groups (*p* > 0.05). Compared to the SHR < 1.14 group, the SHR > 1.45 group had a significantly higher risk of heart failure events (*p* = 0.004), with no signifcant difference observed between the SHR > 1.45 and 1.14 ≤ SHR ≤ 1.45 groups (*p* > 0.05).

### 3.3 Risk factors for MACCEs

The predictors of composite MACCEs were detected by univariate and multivariate regression analysis (Table 2). Using the demographic characteristics, laboratory test results, and hospitalization data as independent variables, and MACCEs as the dependent variable, the univariate analysis identified the following as significantly associated to the occurrence of MACCEs in AMI patients who underwent PCI: SHR, age, heart rate, TyG index, DM, previous PCI, chronic kidney disease, use of antidiabetics, TIMI score, ABG, FPG, HbA1c, *Δ*A-C, hemoglobin, albumin, creatinine, eGFR, EF, pNT-proBNP, and multivessel/left main coronary artery (LMCA). Due to the significant correlation between eGFR and creatinine, creatinine was excluded from the multivariate model. After adjusting for potential confounding factors, such as age, the multivariate analysis identified the following as independent predictors of MACCEs in AMI patients who underwent PCI: SHR, age, albumin, eGFR, EF, and multivessel/LMCA (all, *p* < 0.05).

**Table 2:** Univariate and multivariate analyses and predictors for composite MACCEs.

Variables	Univariate		Multivariate	
	HR (95% CI)	*p*-value	HR (95% CI)	*p*-value
SHR				
SHR < 1.14	Reference		Reference	
1.14 ≤ SHR ≤ 1.45	1.461 (1.167, 1.829)	0.001**	1.488 (1.100, 2.011)	0.010*
SHR > 1.45	2.723 (2.210, 3.355)	<0.001**	2.279 (1.569, 3.311)	<0.001**
Age, years	1.017 (1.011, 1.024)	<0.001**	1.003 (0.996, 1.018)	0.011*
Male gender	1.021 (0.800,1.304)	0.866		
BMI, kg/m^2^	1.011 (0.990, 1.032)	0.317		
SBP, mmHg	1.001 (0.997, 1.004)	0.617		
DBP, mmHg	1.001 (0.995, 1.007)	0.689		
Heart rate, bpm	1.006 (1.001, 1.011)	0.011*	1.003 (0.996, 1.010)	0.386
Current/ex-Smoker	1.000 (0.853, 1.172)	0.999		
STEMI	0.884 (0.758, 1.031)	0.116		
TyG index	1.237 (1.112, 1.376)	<0.001**	1.088 (0.931, 1.271)	0.290
Medical history				
Hypertension	1.165 (0.989, 1.372)	0.067		
Dyslipidemia	0.985 (0.845, 1.148)	0.844		
Diabetes mellitus	1.587 (1.361, 1.850)	<0.001**	1.138 (0.893, 1.450)	0.295
Stoke	1.222 (1.000, 1.494)	0.050		
OMI	1.061 (0.83, 1.358)	0.636		
PCI	1.249 (1.009, 1.545)	0.041*	1.293 (0.980, 1.706)	0.069
CABG	1.602 (0.975, 2.632)	0.063		
Heart failure	1.074 (0.481, 2.401)	0.861		
Chronic kidney diseases	1.643 (1.214, 2.224)	0.001**	1.068 (0.626, 1.823)	0.809
Medication used				
Antiplatelet agent	1.161 (0.982, 1.372)	0.080		
ACEI/ARB	1.057 (0.887, 1.260)	0.533		
Beta-blocker	1.160 (0.932, 1.444)	0.183		
Stain	1.185 (0.953, 1.473)	0.127		
Antidiabetics	1.633 (1.391, 1.918)	<0.001**	1.192 (0.879, 1.616)	0.257
Laboratory values				
WBC, 10^9^/L	1.011 (0.986, 1.037)	0.388		
ABG, mmol/L	1.079 (1.064, 1.095)	<0.001**	1.005 (0.959, 1.052)	0.843
FPG, mmol/L	1.097 (1.073, 1.123)	<0.001**	1.021 (0.974, 1.070)	0.394
HbA1c, %	1.165 (1.117, 1.215)	<0.001**	1.027 (0.925, 1.141)	0.615
ΔA-C, mmol/L	1.098 (1.076, 1.121)	<0.001**		
Hemoglobin, g/L	0.994 (0.988,1.000)	0.037*	1.002 (0.996,1.010)	0.509
Albumin, g/L	0.951 (0.932, 0.970)	<0.001**	0.969 (0.942, 0.997)	0.029*
Creatinine, umol/L	1.002 (1.001, 1.003)	<0.001**		
eGFR, ml/min/1.73 m^2^	0.990 (0.987, 0.994)	<0.001**	0.993 (0.993, 0.988)	0.010*
TC, mmol/L	0.933 (0.865, 1.006)	0.070		
TG, mmol/L	1.004 (0.948, 1.063)	0.895		
LDL-C, mmol/L	0.914 (0.824, 1.013)	0.088		
HDL-C, mmol/L	0.888 (0.647, 1.219)	0.464		
D-Dimer, ug/ml	1.016 (0.999, 1.034)	0.073		
Uric acid, umol/L	1.001 (1.000,1.002)	0.062		
hs-CRP, mg/L	1.000 (0.998, 1.002)	0.813		
EF, %	0.204 (0.100, 0.416)	<0.001**	0.370 (0.159, 0.863)	0.021*
pNT-proBNP, pg/ml	1.000 (1.000, 1.000)	<0.001**	1.000 (1.000, 1.000)	0.085
pMyo, ng/ml	1.000 (1.000, 1.001)	0.268		
pCK-MB, ng/ml	1.000 (0.999, 1.000)	0.643		
pTNI, ng/ml	1.001 (0.996, 1.007)	0.597		
Angiography findings				
Multivessel/LMCA	1.355 (1.030, 1.783)	0.030*	1.435 (1.094,1.884)	0.009**
Proximal LAD	0.973 (0.801, 1.182)	0.783		
TIMI score	1.058 (1.005, 1.115)	0.033*	0.984 (0.932,1.040)	0.579
Hospitalization indicators				
Primary PCI	0.987 (0.843, 1.156)	0.875		
Antiplatelet agent	0.854 (0.624, 1.167)	0.322		
ACEI/ARB	0.955 (0.812, 1.123)	0.578		
Beta-blocker	0.957 (0.806, 1.137)	0.620		
Stain	0.992 (0.786, 1.251)	0.943		
Antidiabetics	1.627 (1.389, 1.905)	<0.001**	1.166 (0.864, 1.573)	0.315

MACCEs, major adverse cardiac and cerebrovascular events; HR, hazard ratio; CI, confidence interval; SHR, stress hyperglycemia ratio; BMI, body mass index; SBP, systolic blood pressure; DBP, diastolic blood pressure; STEMI, ST-segment elevation myocardial infarction; TyG, triglyceride-glucose; OMI, old myocardial infarction; PCI, percutaneous coronary intervention; CABG, coronary artery bypass graft; ACEI/ARB, angiotensin-converting enzyme inhibitor/angiotensin receptor blocker; LMCA, left main coronary artery; LAD, left anterior descending; TIMI, thrombolysis in myocardial infarction; WBC, white blood cells; ABG, admission plasma glucose; FPG, fasting plasma glucose; HbA1c, glycated hemoglobin; ΔA-C, ABG-(1.59 × Hb A1c%−2.59); eGFR, estimated glomerular filtration rate; TC, total cholesterol; TG, triglyceride; LDL-C, low-density lipoprotein cholesterol; HDL-C, high-density lipoprotein cholesterol; hs-CRP, high-sensitivity C reactive protein; LVEF, left ventricular ejection fraction; pNT-proBNP, peak of N-terminal pro-brain natriuretic peptide; pCK-MB, peak of creatine kinase isoenzyme MB; pTNI, peak of cardiac troponin I.

* *p* < 0.05.

** *p* < 0.01.

The unadjusted competing risk modeling revealed that the cumulative incidence of all-cause mortality, CV death, recurrent MI, target vessel revascularization, and readmission for heart failure significantly increased in the SHR > 1.45 group (all, *p* < 0.001). After adjusting for potential confounders, such as age and TyG, the multivariate-adjusted hazard ratio (HR) for target vessel revascularization remained significantly higher in patients with SHR >1.45 (*p* < 0.05, Table 3).

**Table 3:** Univariate and multivariate analyses and predictors of various cardiovascular events.

Cardiovascular events	Events (%)	Unadjusted HR (95%)	*p*-value	Adjusted HR (95% CI)	*p*-value
All-cause death					
SHR < 1.14	64 (6.03)	Reference	−/−	Reference	−/−
1.14 ≤ SHR ≤ 1.45	102 (9.29)	1.295 (0.946, 1.773)	0.107	1.091 (0.743, 1.603)	0.655
SHR > 1.45	124 (11.78)	1.947 (1.439, 2.634)	<0.001**	1.397 (0.861, 2.265)	0.176
CV death					
SHR < 1.14	29 (2.73)	Reference	−/−	Reference	−/−
1.14 ≤ SHR ≤ 1.45	47 (4.28)	1.365 (0.858, 2.170)	0.189	1.472 (0.802, 2.704)	0.212
SHR > 1.45	70 (6.65)	2.424 (1.572, 3.738)	<0.001**	1.727 (0.823, 3.626)	0.149
Recurrent MI					
SHR < 1.14	46 (4.34)	Reference	−/−	Reference	−/−
1.14 ≤ SHR ≤ 1.45	74 (6.74)	1.376 (0.952, 1.990)	0.090	1.281 (0.816, 2.011)	0.281
SHR > 1.45	96 (9.12)	2.117 (1.489, 3.009)	<0.001**	1.714 (0.94, 3.127)	0.079
Target vessel revascularization					
SHR < 1.14	66 (6.22)	Reference	−/−	Reference	−/−
1.14 ≤ SHR ≤ 1.45	99 (9.02)	1.324 (0.969, 1.808)	0.078	1.255 (0.901, 1.749)	0.180
SHR > 1.45	153 (14.53)	2.354 (1.764, 3.142)	<0.001**	1.998 (1.299, 3.074)	0.002**
Readmission for heart failure					
SHR < 1.14	21 (1.98)	Reference	−/−	Reference	−/−
1.14 ≤ SHR ≤ 1.45	37 (3.37)	1.588 (0.929, 2.715)	0.091	1.500 (0.833, 2.704)	0.177
SHR > 1.45	51 (4.84)	2.477 (1.490, 4.119)	<0.001**	1.896 (0.916, 3.926)	0.085
Stoke					
SHR < 1.14	16 (1.51)	Reference	−/−	−/−	−/−
1.14 ≤ SHR ≤ 1.45	20 (1.82)	0.985 (0.509, 1.906)	0.964	−/−	−/−
SHR > 1.45	20 (1.82)	1.449 (0.765, 2.744)	0.255	−/−	−/−

The adjusted factors included the following: age, heart rate, triglyceride-glucose index, history of diabetes mellitus, percutaneous coronary intervention, chronic kidney diseases before admission, antidiabetics used, angiography findings (multivessel/left main coronary artery), admission plasma glucose, fasting plasma glucose, glycated hemoglobin, white blood cells, albumin, estimated glomerular filtration rate, left ventricular ejection fraction, and peak of N-terminal pro-brain natriuretic peptide. HR, hazard ratio; CI, confidence interval; SHR, stress hyperglycemia ratio; CV, cardiac death; MI, myocardial infarction.

** *p* < 0.01.

### 3.4 Independent associations of SHR with MACCEs in various subgroups

**Figure 4:**
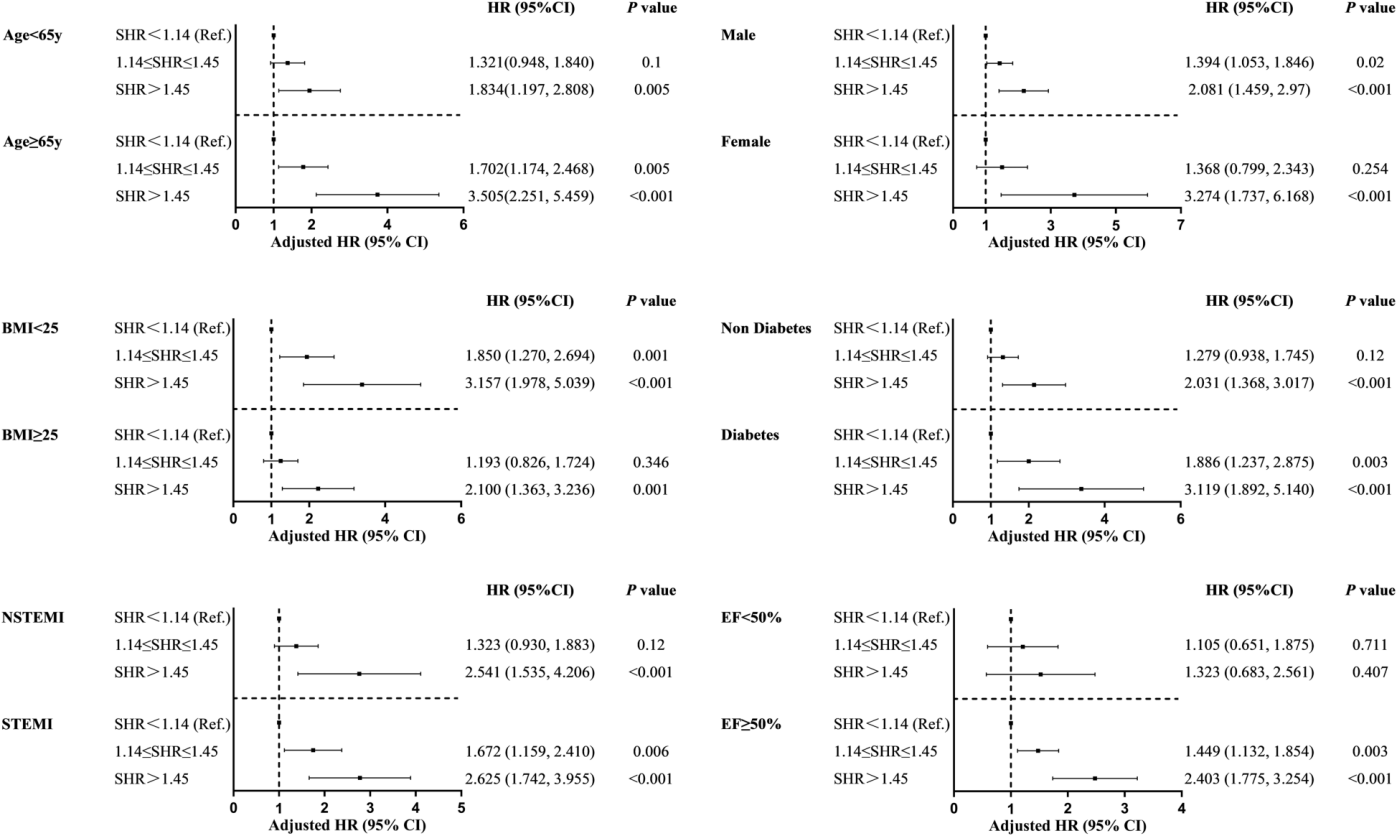
Forest plot for composite MACCEs in the different subgroups.

A subgroup analysis was conducted based on age, gender, DM, LVEF, BMI, and type of AMI (Figure 4). The results were generally consistent with the overall analysis results, except for the subgroup with EF <0.5. For all other groups, SHR predicted the occurrence of MACCEs during the follow-up period (*p* < 0.05). This suggests that the prognostic value of SHR was not significantly affected by the key factors correlated to AMI.

### 3.5 ROC curve analysis of SHR

**Figure 5:**
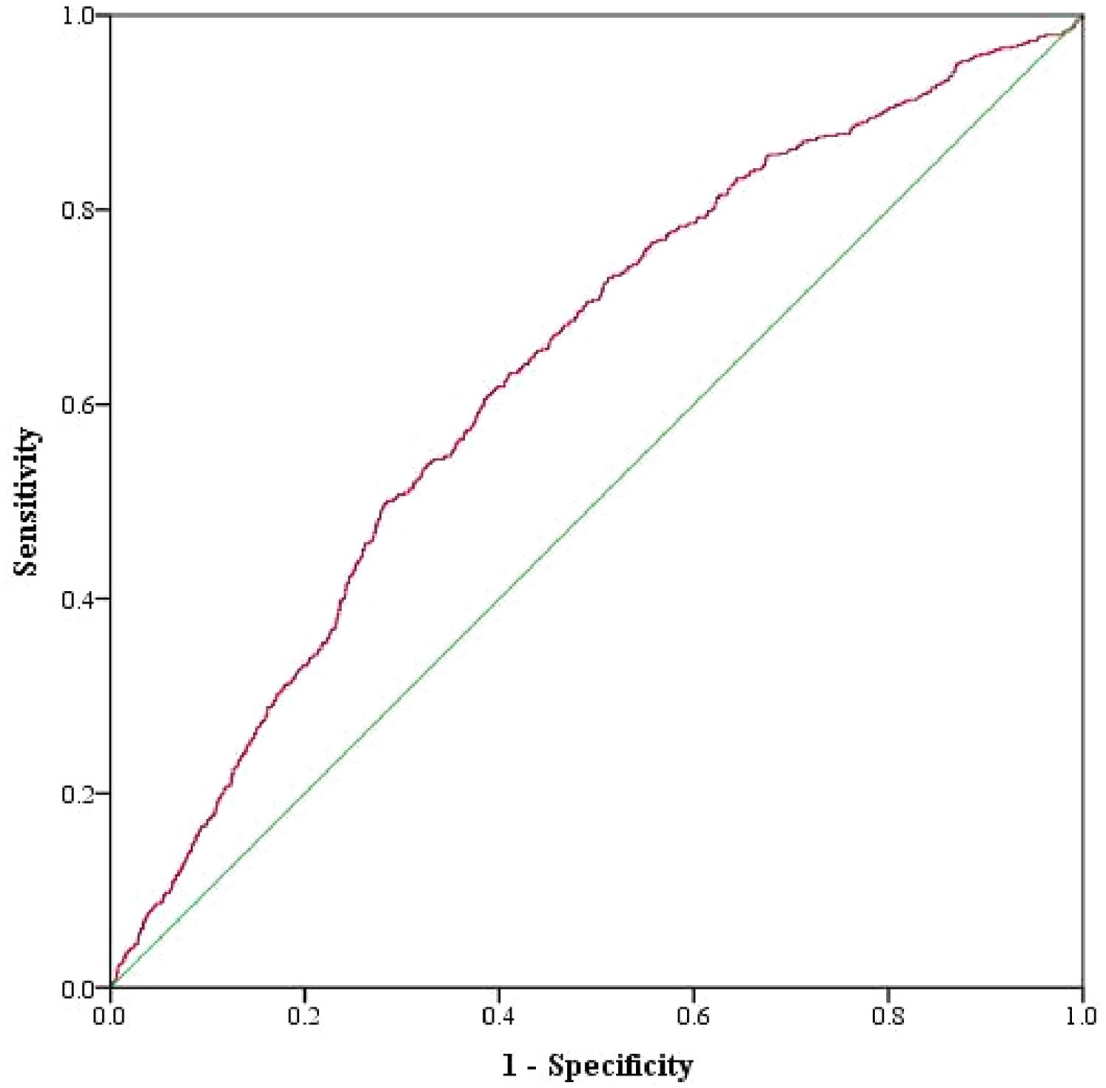
ROC curves for SHR, as a predictor of composite MACCEs, in AMI patients who underwent PCI.

The predictive efficacy of SHR for MACCEs during the follow-up period was determined by ROC curve analysis. The area under the ROC curve was 0.636 (95% CI: 0.613–0.659, *p* < 0.05), indicating that SHR has significant predictive value for follow-up MACCEs. The optimal cut-off value for SHR in predicting MACCEs was 1.317, with a Youden's index of 0.221, a sensitivity of 0.631, and a specificity of 0.591 (Figure 5).

## 4 Discussion

The present study investigated the prognostic value of SHR for cardiovascular events in AMI patients who underwent PCI, including both STEMI and non-STEMI. The present main findings were, as follows: (1) the incidence of MACCEs was significantly correlated to the increase in SHR; (2) SHR was an independent predictor of MACCEs in AMI patients who underwent PCI, which included all-cause mortality, CV death, recurrent MI, revascularization, heart failure, and composite MACCEs; (3) in terms of gender, age, type of AMI, BMI, LVEF, and DM, SHR >1.45 was significantly correlated to MACCEs in all subgroups, except for EF <50%; (4) the area under the ROC curve for SHR in predicting MACCEs was 0.636, with a cut-off value of 1.317; (5) age, albumin, eGFR, EF, and multivessel/LMCA were the independent predictors of MACCEs in AMI patients who underwent PCI.

The activation of sympathetic nerves after AMI induces the release of glucagon, growth hormones, glucocorticoids and catecholamine, leading to the increase in blood glucose through the promotion of gluconeogenesis, accelerating the liver glycogen breakdown, and reducing the insulin uptake of glucose. Stress hyperglycemia has been identified as a key risk factor for cardiovascular diseases and adverse clinical outcomes (9, 19). The harm of stress hyperglycemia on AMI patients is mainly correlated to metabolic disorders, inflammation, hypercoagulability, oxidative stress, and endothelial dysfunction. MI patients with hyperglycemia exhibited an increase in left ventricular dysfunction, and a broader range of myocardial necrosis, as evidenced by the wider extent of microvascular obstruction and late gadolinium enhancement observed on the cardiac magnetic imaging, indicating a positive correlation between admission hyperglycemia and MI (20). The adverse effect of stress hyperglycemia on patient prognosis is independent of the DM itself. Elevated SHR is associated to increased risk of heart failure progression in patients with significant secondary mitral regurgitation after PCI, particularly in patients with normoglycemia (21). For MI patients without diabetes, the stress hyperglycemia on admission was associated to the increase in in-hospital death and late-follow-up MACCEs (i.e., stroke, recurrent MI, and all-cause mortality). In addition, stress-induced glycemic increase, older age, and low baseline LVEF predicted the MACCEs during the follow-up period (22). Furthermore, elevated SHR independently predicted the poor short- and long-term outcomes in patients with acute heart failure (23). Cui et al. reported that high ABG is positively correlated to 2-year mortality in patients with AMI complicated by DM, prediabetes, and normal glucose regulation. Stress hyperglycemia can be a useful marker for risk stratification, in both diabetic patients and subjects with normal glucose regulation. However, the ABG threshold needs to be adjusted according to different glucose metabolism statuses (24). Compared to a single blood glucose measurement, SHR provides better adjustment for the effects of the last meal and basal blood glucose, reflects the relative increase in blood glucose, and helps to identify patients with true stress hyperglycemia. Therefore, SHR can be used to identify stress hyperglycemia in clinical practice.

Extensive research has demonstrated the predictive value of SHR in AMI patients. A meta-analysis and systematic review of 32 studies suggested that elevated SHR is associated to increased all-cause mortality risk in hospitalized patients with AMI or acute ischemic stroke (25). In a retrospective study that analyzed 905 patients diagnosed with STEMI, the incidence of no-reflow increased with the increase in SHR levels in STEMI patients who underwent emergency PCI, regardless of the DM status (26). In a study conducted on coronary artery disease patients who underwent PCI with a median follow-up of 2.5 years, the highest SHR quartile group had significantly higher risk of MACCEs in the overall population (HR: 1.31, 95% CI: 1.05–1.64), and in non-diabetic patients (HR: 1.45, 95% CI: 1.02–2.06), when compared to the lower three quartiles. SHR can effectively predict the occurrence of MACCEs after PCI, particularly in STEMI patients without DM (27). Wei et al. reported that SHR is independently correlated to risk of mortality and major adverse cardiovascular events in patients with STEMI. Furthermore, incorporating SHR into the Thrombolysis in Myocardial Infarction risk score may improve its predictive accuracy for STEMI patients, especially for patients with DM (28). In the present study, it was found that SHR independently predicted the occurrence of MACCEs in AMI patients who underwent PCI, which is consistent with the aforementioned studies. In addition, a study conducted on 5,562 patients revealed that ACS patients who received drug-eluting stent implantation had the lowest incidence of MACCEs in the third quintile of SHR, according to the Kaplan–Meier survival analysis results. Furthermore, SHR exhibited a U-shaped relationship with the incidence rates of MACCEs and major adverse cardiovascular events after the 2-year follow-up period, and a J-shaped relationship with in-hospital CV death and MI incidence after the 2-year follow-up period (3). In the present study, there was no significant difference in cumulative incidence of cardiovascular events between the two groups with lower SHR tertiles, and no corresponding phenomenon was observed. However, it was suggested that the sample size should be expanded, and the groupings should be refined to further verify the findings.

The present study revealed that the cumulative incidence of other MACCEs significantly increased with the increase in SHR, except for stroke. However, SHR did not independently predict the occurrence of cardiovascular events, which may be correlated to the small number of follow-up events, and the limited follow-up time. The novel aspect of the present study was that the predictive value of SHR for MACCEs in various subgroups was analyzed, which included BMI, gender, age, presence or absence of DM, LVEF, and type of AMI. The results revealed that SHR effectively predicted the MACCEs in most subgroups, except for patients with EF <50%. In a meta-analysis conducted for a heart failure cohort, elevated SHR had an increased risk ratio for all-cause mortality, although the confidence interval was wide, and included the null [RR 1.34 (95% CI: 0.89–2.01), *p* = 0.17] (25).

In the present study, among patients with EF <50%, the rates of primary PCI (*p* < 0.001) and antidiabetic therapy (*p* = 0.002) were higher in patients in the SHR > 1.45 group, when compared to the other groups, with no differences in other treatments. This was consistent with the overall population. A study conducted on 2,596 patients with MI revealed that despite the advancements in the epidemiology and management of MI, the mortality rate for heart failure patients remained high at 70% during an average follow-up of 7.6 years (29). After adjusting for age and gender, heart failure was identified to have a strong association to mortality, as a time-dependent variable. Therefore, it can be reasonably considered that patients with heart failure have a significantly poor prognosis, and that a high SHR would not further exacerbate the poor prognosis.

The present study has several limitations. First, despite the large sample size, the present study had a single-center design. Thus, the findings need to be verified through multi-center and larger cohort studies. Second, the present study excluded patients with missing data, and patients who were lost to follow-up, which may have introduced selection bias. Third, a recent study has demonstrated for the first time that periprocedural myocardial injury, especially type 4a MI, is associated with a significant worse prognosis (30). However, in this study, reinfarction did not include periprocedural myocardial infarctions, which may have resulted in the loss of valuable prognostic information. Forth, due to the retrospective observational nature of the present study, further explorations with a prospective design on the effects of stress hyperglycemia control in reducing long-term MACCEs are needed. As an emerging non-invasive marker, SHR deserves greater clinical attention. Although no randomized trials have been conducted to date, large observational studies have highlighted its relevance in real-world cardiovascular disease management and stress hyperglycemia intervention. Future research should focus in clarifying the diagnostic utility of SHR in cardiovascular disease, optimizing its integration into cardiovascular risk assessment, and exploring its potential as a therapeutic target.

## 5 Conclusions

The present study confirms that stress hyperglycemia, as expressed by SHR, is a strong independent predictor of MACCEs in AMI patients undergoing PCI. These findings provide important guidance for clinicians in predicting follow-up clinical events in patients with AMI.

## Data Availability

The raw data supporting the conclusions of this article will be made available by the authors, without undue reservation.
